# Biomechanical behavior of immediately placed implant using bone graft and socket shield techniques: a 3D finite element analysis

**DOI:** 10.1186/s13005-025-00537-2

**Published:** 2025-08-13

**Authors:** Reham A. Rashwan, Sanaa H. AbdElkader, Noha M. Elkersh, Rewaa G. AboElhassan

**Affiliations:** 1 Department of Conservative Dentistry, Division of Fixed Prosthodontics, Faculty of Dentistry, Alexandria University, Alexandria, Egypt; 2 Department of Oral Medicine, Periodontology, Oral Diagnosis and Oral Radiology, Faculty of Dentistry, Alexandria University, Alexandria, Egypt

**Keywords:** Finite element analysis, Immediate implant, Immediate load, Bone graft, Socket shield

## Abstract

**Background:**

Recently, several techniques for immediate implant placement have gained popularity, offering numerous advantages. These include the preservation of bone around the tooth socket and enhanced aesthetics. Nonetheless, the biomechanical behavior of implants and peri-implant tissues under immediate loading with these techniques remains uncertain. This study examines stress distribution surrounding an immediately placed implant, using socket shield and bone graft techniques compared to a healed socket.

**Materials and methods:**

Cone-beam computed tomography (CBCT) scans of the anterior maxilla were used to construct finite element analysis (FEA) models. The process of modeling the implant, abutment, and provisional crown used standard tessellation language (STL) files of the original components. The implant was modeled in three clinical scenarios: a healed socket (HS), an extraction socket with bone graft (BG), and a socket shield (SS). A frictional contact (µ = 0.3) was established to simulate immediate loading. An axial load of 25.5 N and a non-axial load of 178 N at a 30° angle were applied along the implant’s long axis in a palatal direction. FEA was conducted for stress distribution analysis.

**Results:**

In evaluating maximum principal, von Mises stress distribution within the cortical bone, the HS model exhibited the highest stress level, with a maximum of 125 MPa, 127 MPa, respectively. The SS model demonstrated the lowest stress, recording a maximum of 82 MPa, 90.7 MPa, respectively, while the BG model had a maximum value of 115 MPa, 116.84 MPa, respectively. When assessing the von Mises stress distribution associated with the implant, the HS model recorded the highest stress value of 385 MPa. In contrast, the BG and SS models recorded lower stress values of 252 MPa and 281 MPa, respectively.

**Conclusions:**

The socket shield technique exhibits advantageous biomechanical performance under immediate loading conditions by reducing stress on peri-implant bone and implant components. These results endorse its clinical applicability but necessitate further in vivo validation.

## Introduction

Dental implants are a widely recognized and effective therapeutic option for partially edentulous patients, demonstrating sustained efficacy in both the mandible and maxilla. The quality and quantity of bone significantly influence the osseointegration of dental implants with the surrounding bone tissue [1]. However, the loss of the periodontal ligament, along with the stress experienced in the buccal bone plate, which tends to undergo more significant resorption compared to the lingual and palatal regions of the alveolus, is associated with alterations in the bony ridges following tooth extractions. This resorption might necessitate additional tissue reconstructions or lead to modest aesthetic outcomes, potentially complicating dental implant insertion [2]. 

Immediate implant placement has emerged as a leading technique in recent years, offering many benefits. These include preserving the bone surrounding the tooth socket and enhancing aesthetics. Moreover, it mitigates the disadvantages of delayed implants, including extended treatment duration and alveolar bone resorption. However, a key challenge to immediate implantation is the jumping gap between the implant’s coronal end and the socket wall. This gap arises due to a dimensional discrepancy between the extraction socket and the implant. When this distance exceeds 2 mm, bone defect formation can occur. Consequently, the stability of the implant is significantly reduced [3]. To address the challenge of residual space between the extraction socket and the implant, experts have recommended incorporating barrier materials and bone grafts. These materials facilitate the regeneration of lost bone and are essential for maintaining the structural integrity of both hard and soft tissues [4]. A range of regenerative biomaterials, including growth-promoting factors and bone grafts such as autogenous grafts, xenografts, and alloplastic grafts, have been investigated to help preserve the volume of the alveolar ridge following tooth extractions [4]. Autogenous bone is considered the gold standard. However, one of the significant limitations of using autologous bone is the necessity of a secondary surgical procedure to harvest the graft [5].; A xenograft refers to bone tissue sourced from non-human organisms, with deproteinized bovine bone mineral—for instance, Bio-Oss^®^, manufactured by Geistlich Pharma AG in Wolhusen, Switzerland—being a common example. In contrast, alloplastic graft materials are synthetic bone substitutes not derived from living organisms [6]. 

In 2010, Hürzeler et al. introduced the socket shield technique to preserve the buccal bone plate and enhance the predictability of aesthetic outcomes. This technique consists of several key steps, including the removal of the coronal part, splitting the root in a mesiodistal direction, extracting the lingual fragment, retaining the buccal root fragment in contact with the buccal socket wall, and then immediate implantation of the dental implant [7]. 

Cherel and Etienne [8], Kan and Rungcharassaeng [9], and Tan et al. [10] have demonstrated that the SS technique can safeguard the mesial, distal, and buccal bone plates during immediate implantation. This approach highlights the potential benefits of maintaining a root fragment to enhance bone preservation in dental implants.

Various classifications have been used to determine the timing between implant placement and loading. The terms immediate, progressive, early, and delayed loading describe different implant loading protocols. For several years, delayed loading was implemented to mitigate the risk of implant failure resulting from movements at the interface.

However, it was observed that there were high implant failure rates due to fibrous encapsulation around the implants. Improvements in surgical technique, implant design, and masticatory forces have shown that immediate loading procedures can achieve successful outcomes. Over the past decade, the high predictability of immediate placement and restoration/loading protocols has led to a bimodal approach, where immediate implant placement is combined with a single crown after implantation. The primary advantage of this bimodal approach is that it combines alveolar bone preservation through immediate implantation with peri-implant mucosal preservation following immediate restoration/loading. This protocol reduces papilla loss and buccal or palatal recession of the peri-implant mucosa. Additionally, it offers the shortest treatment time among other protocols, which is vital in anterior aesthetic regions [11]. 

One of the critical aspects of dental implants is that the implant-bone interface lacks a shock absorption mechanism, resulting in the complete transmission of occlusal loading forces onto the interface without any mitigation [12]. Excessive mechanical loading on the bone-implant interface (BIC) is a significant factor contributing to the development of peri-implantitis, which can ultimately result in implant failure [13]. The impact of mechanical stimuli on dental implant performance and Osseointegration upon immediate loading is not fully understood [14]. However, understanding the biomechanical interactions between the implant and surrounding bone is essential to maintain the dental implant’s integrity and longevity. A critical aspect of this understanding involves analyzing stress distribution around the implants. FEA is a practical approach for evaluating stress and strain distribution in peri-implant bone [15]. Its applications in assessing various parameters within the peri-implant region are extensive, encompassing immediate loading, stress, and strain distribution [16]. 

Earlier research assessed stress distribution on anterior implants and peripheral bone through FEA [17–19]. However, insufficient literature addresses the biomechanical response and stress generation related to implant placement techniques under immediate loading. Therefore, our study simulates immediate loading through provisional restoration to evaluate the biomechanical performance of immediately placed implants using bone graft (BG) and socket shield (SS) techniques compared to healed socket (HS). The topic is clinically relevant, particularly given the increasing interest in optimizing post-extraction implant protocols. Highlight how the findings can guide clinical decision-making in immediate loading situations.

The study’s rationale was that socket shield and bone graft techniques might affect the stress distribution on the immediately placed implant and the surrounding osseous structures. The null hypothesis was that the socket shield technique transmits forces to the immediately placed implant and the surrounding osseous structures, similar to the bone graft technique.

## Materials and methods

This in vitro study was conducted at the Conservative Department of the Faculty of Dentistry, Alexandria University, and received approval from the Research Ethics Committee. The approval code of this study is (Ethics code number: 001056– IORG 0008839). The purpose of this in vitro investigation was to simulate immediate loading through provisional restoration to evaluate the biomechanical performance of immediately placed implants in three distinct clinical scenarios, including a healed socket (HS), an extraction socket with a bone graft (BG), and a socket shield technique (SS). The study employed finite element analysis software to analyze the stress distribution.

**Methodology**

**Steps of simulation**:

1. Model Generation.
2. Assembly of parts.
3. Defining materials’ mechanical properties.
4. Mesh generation.
5. Boundary conditions and load application.
6. Finite element analysis processing.

### Model generation

The bone model was created by reconstructing previously obtained anonymized CBCT data (Vatech Green X, 90 kV, 0.12 mm voxel) with ethical approval obtained from our institutional review board (Ethics code: 001056– IORG 0008839). CBCT was saved in Digital Imaging and Communications in Medicine (DICOM) format, exported, and superimposed on a reverse engineering program, Mimics (Materialise, Belgium, version 19.0), converting the scanned data into a complete 3D Computer-Aided Design (CAD) model. Subsequently, the model’s standard tessellation language (STL) file was exported into 3D modeling software SpaceClaim (ANSYS Workbench 19.0) for further editing or modeling.

The bone model was simulated by an outer layer of 2-mm-thick cortical bone and an inner layer of high-density cancellous bone, which is presumed to exhibit optimal bonding properties. This represents D2 bone tissue commonly observed in the anterior maxilla [20, 21]. Geometric simplification in mesiodistal extension and buccal contouring of the bone model was made to reduce computational complexity and allow controlled variable comparison across models.

Using the same 3D modeling software, a 4 mm × 10 mm titanium implant (Megagen AnyRidge) and EZ Post Abutment were modeled. After creating the implant and the abutment, a provisional PMMA crown (7.5 mm height, 1.5 mm marginal thickness) was designed to fit over the standard titanium abutment as a provisional restoration.

### Assembly of parts

**Fig. 1:**
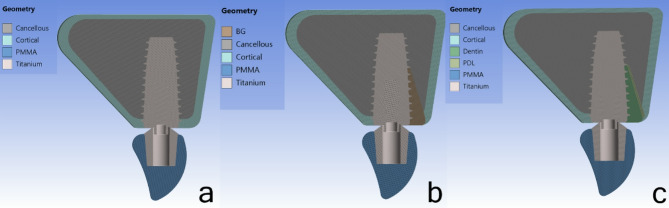
Scheme of the implant placement techniques and experimental models. **a**: HS model; **b**: BG model; **c**: SS model

Different components of the model, including the implant, abutment, provisional crown, and bone, were positioned adjacent to each other in three clinical scenarios: a healed socket (HS), an extraction socket with a bone graft (BG), and a socket shield technique (SS). Figure 1.

Model (HS): The implant was simulated to be placed entirely within the osseous tissue of a healed socket, simulating both cortical and cancellous bone.

Model (BG): The implant technique was simulated immediately after the maxillary incisor was extracted. A bone graft (Bio-Oss, Geistlich, Wolhusen, Switzerland) was placed in the jumping gap. It was assumed that the simulated bone graft sufficiently filled the jumping gap.

Model (SS): The socket shield technique was simulated during tooth extraction. This technique preserved a 1.5-mm-thick and 6-mm-long labial root fragment and its ligament. It was assumed that the implant’s buccal threads were in contact with the labial root fragment.

### Defining materials’ mechanical properties

The mechanical properties, including Poisson’s ratios and elastic moduli, for each component in the three models were derived from either manufacturer data or earlier research. These properties are summarized in the Table 1 [17, 22–24]. All components used in the three models were assumed to be linearly elastic, isotropic, and homogeneous.

**Table 1:** Poisson’s ratios and elastic moduli for each component in the three models [17, 22–24]

Material	Elasticity Modulus (E)	Poisson’s Ratio (*n*)
Cortical bone	13.7GPa	0.3 [24]
High-density Cancellous bone	1.37GPa	0.3 [24]
Bio-Oss	0.36GPa	0.3 [22]
Titanium implant	110GPa	0.35 [17]
Titanium abutment	110GPa	0.35 [17]
Dentin	18.6GPa	0.31 [24]
Periodontal ligament	0.07GPa	0.45 [23]
PMMA	2.7GPa	0.35 [23]

### Mesh generation

The CAD models were imported into the preprocessing software, ANSYS Workbench 19.0 (Canonsburg, PA, Analysis Inc., Houston, USA), for modeling and solid mesh generation. This process involved reconstructing three-dimensional solid models in STP format.

A mesh convergence test was conducted to optimize the balance between computational efficiency and result reliability. Three mesh densities were tested until the stress values converged with an error of less than 5%, indicating sufficient convergence. The finite element mesh was standardized to use 0.3 mm linear tetrahedral solid elements and their associated nodes. Each model included a specific number of structures, nodes, and elements, as detailed in Table 2.

**Table 2:** Number of structures, nodes, and elements in each model

Model	Structures	Nodes	Elements
SS	8	343,214	218,010
BG	7	340,228	216,952
HS	6	330,620	211,551

### Boundary conditions and load application

**Fig. 2:**
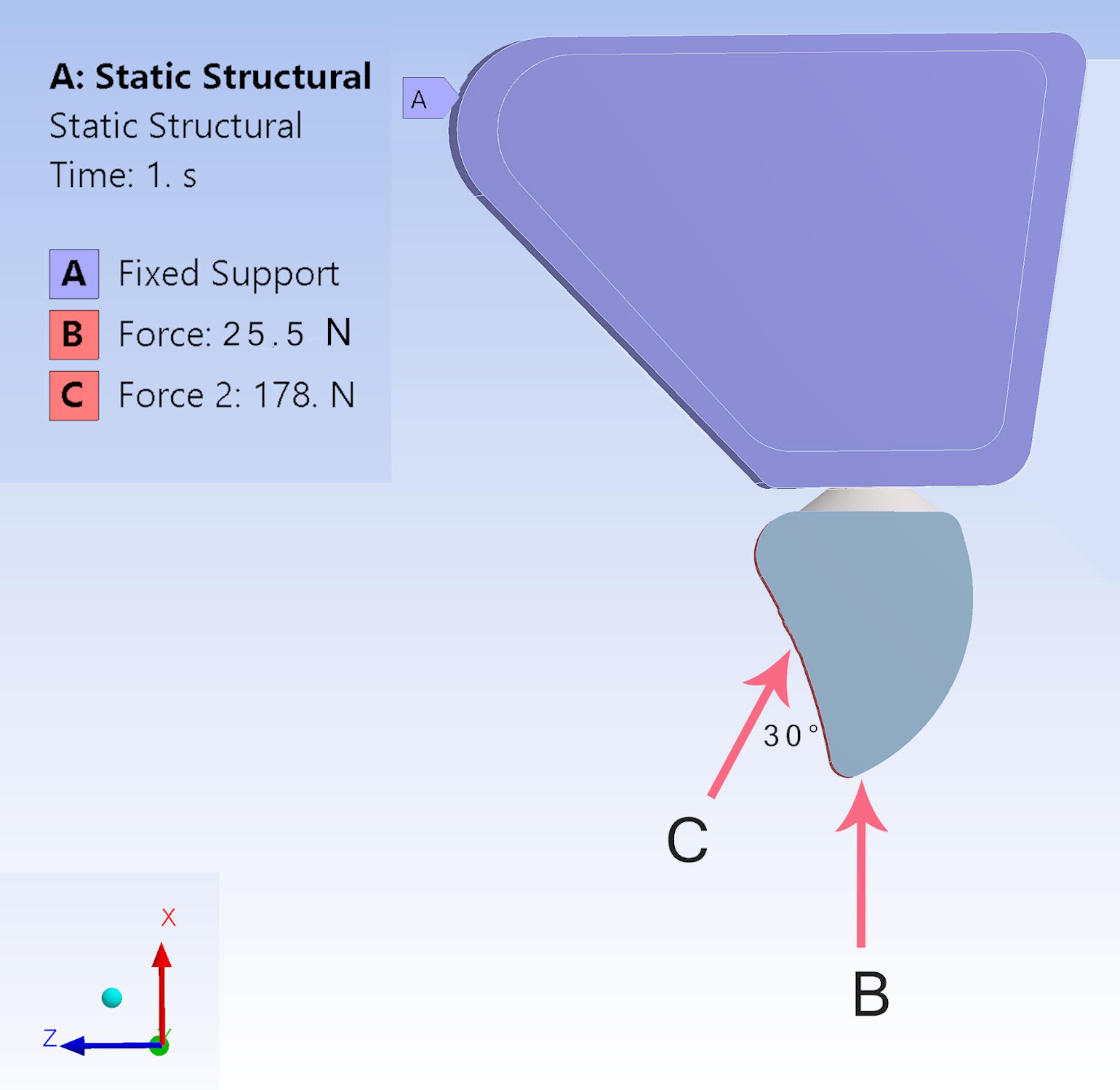
Side view of **A**: bone tissue as fixed support, **B**: 25.5 N loads perpendicularly applied to the implant long axis, **C**: oblique 178 N, and N: Newton

Boundary conditions are essential in FEA, as they reflect the movements at the nodes and their corresponding relationships. The following factors were considered for all models:

1. In all models, the abutment/implant, abutment/provisional crown interface was assumed to be perfectly bonded [25, 26].
2. All models were subjected to rigid fixation, effectively restricting all degrees of freedom at the nodal points located in the upper and distal regions of cortical and trabecular bone. This methodology prevented any movement across the x, y, and z axes, ensuring stability and accuracy in the observations.
3. Immediate loading was simulated by setting the friction coefficient to 0.3 as non-osseointegrated between Bone graft/bone, implant/bone, implant/dental fragment, and implant/bone-graft. The contact type between the dental fragment/bone was assumed to be bonded. Table 3 [17, 19, 27–29].
4. The preload resulting from the applied tightening torque was quantified at 522 N [30]. This stress was incorporated into the analyses as the initial load step.
5. This study employed an axial load of 25.5 N perpendicular on the long axis of the implant to simulate protrusive movements. Additionally, a non-axial 178 N force was applied at a 30° angle to the palatal surface of the PMMA crown, representing natural clinical conditions in the anterior region [31, 32].
6. The software conducted a combined analysis of the two loads applied simultaneously, representing mixed loading conditions [18, 33]. Figure 2.

**Table 3:** The friction coefficient between each component in the three models [17, 19, 27–29]

Component	Friction coefficient
Implant/Bone	0.3 [17]
Implant/Bone-graft	0.3 [27]
Implant/Dental fragment	0.3 [19]
Bone graft/Bone	0.3 [28]
Dental fragment/Bone	Bonded [19, 29]

**Table 4:** The peak values of von mises stress in the implant, abutment, and crown

von Mises stress (MPa)	HS	BG	SS
Implant	385	252	281
Abutment	936	505	524
Crown	37	38	40
Cortical bone	127	116.84	90.73
Cancellous bone	8.76	4.8	5.3
Graft / Dentin	Not applicable	3.7	29.5

**Table 5:** The peak values of maximum principal stress in cortical, cancellous, bone graft, and dentin

Maximum principal stress (MPa)	HS	BG	SS
Cortical bone	125	115	82
Cancellous bone	7.6	3.6	4.3
Graft / Dentin	Not applicable	2.3	28.3

### Finite element analysis processing

This study utilized ANSYS Workbench 19.0 (Canonsburg, PA, Analysis Inc., Houston, USA) for computer-aided mechanical processes. A nonlinear static FEA was conducted to determine the stress distribution patterns across various models.

Nonlinear static FEA is a non-time-dependent analysis that accounts for nonlinearities such as geometric, material, and contact behaviors. In our study, we included contact nonlinearity, essential for evaluating the structural performance of implants and prosthetic components in immediate loading scenarios [34]. Von Mises stress was analyzed to understand the biomechanical performance of the implant, abutment, and peri-implant tissues. Since von Mises stress combines the effects of tensile, compressive, and shear stresses, it offers a comprehensive view of overall stress levels at any point. This approach is especially valuable in implant-supported restorations, where complex multi-directional stresses occur due to oblique loading. Additionally, von Mises stress provides a more precise visualization of stress concentration areas and is commonly utilized in research [19, 25, 26]. Its use in this study provides a practical, clinically relevant way to assess the risk of peri-implant bone overloading. Max principal stress was analyzed to understand the biomechanical behavior of peri-implant tissues and to evaluate the risk of tensile failure in bone, which acts as a brittle material in tensile areas.

Failure occurs when the stresses meet or exceed the material’s ultimate and yield strength. The ultimate strength of bone is influenced by direction, location, and individual variations. The ultimate tensile stress of cortical bone ranges from 121 to 135 MPa, and the ultimate compressive stress ranges from 167 to 205 MPa. In contrast, the strength of cancellous bone is significantly lower than that of cortical bone, with ultimate stress values ranging from 1 to 20 MPa [35]. 

These results were visually represented through a graphical user interface, displaying the outcomes. For each material, results were illustrated as color-coded diagrams, where similar colors indicate equivalent stress ranges and warmer colors denote higher stress levels [36]. 

## Results

**Fig. 3:**
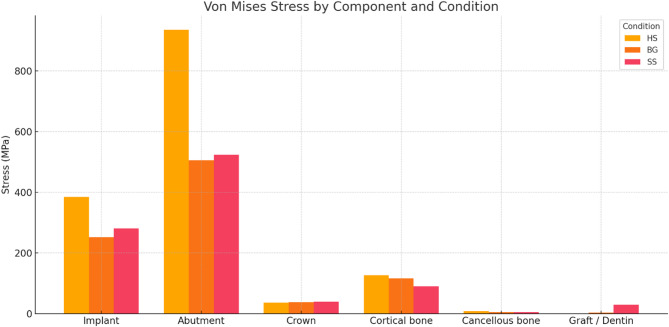
The peak values of von Mises stress in different structures and models

**Fig. 4:**
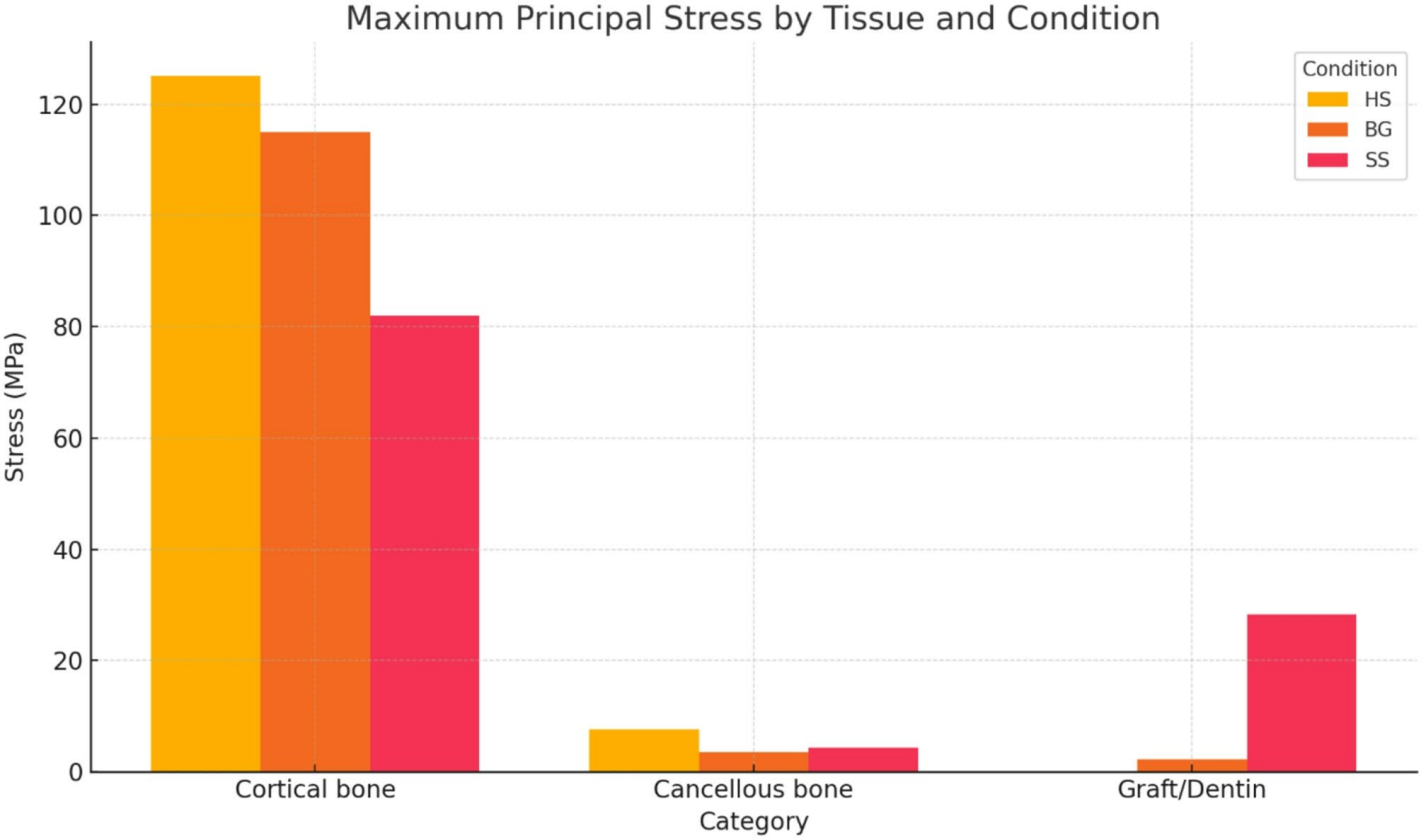
The peak maximum principal stresses in different structures and models

Finite Element Analysis (FEA) of models simulating three clinical scenarios involving immediately loaded implants in the pre-maxilla—specifically, healed socket (HS), extraction socket with bone graft (BG), and socket shield technique (SS)—was visualized and calculated using shaded images to illustrate stress distribution. The red zone represents the highest stress levels, whereas the dark blue zone signifies the lowest. Stress values that differ by less than 5% are regarded as similar. The stress levels were calculated using the von Mises stress in the implant, abutment, and peri-implant tissues and the maximum principal stress in the peri-implant tissues. The peak values of von Mises and max principal stresses observed in different structures and models are described in the Table 4, 5; Figs. 3 and 4.

**Fig. 5:**
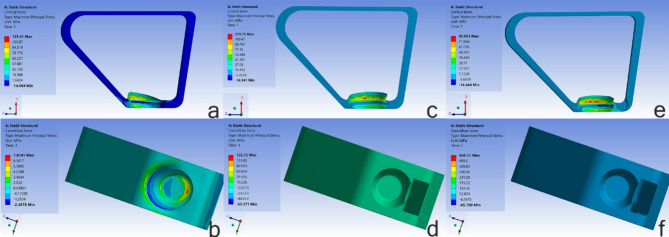
Maximum principal stress distribution in each model’s cortical bone, cancellous bone. MPa: Mega Pascal; Min: Minimum; Max: Maximum, **a**,** b** in HS model; **c**,** d** in BG model; **e**,** f** in SS model

**Fig. 6:**
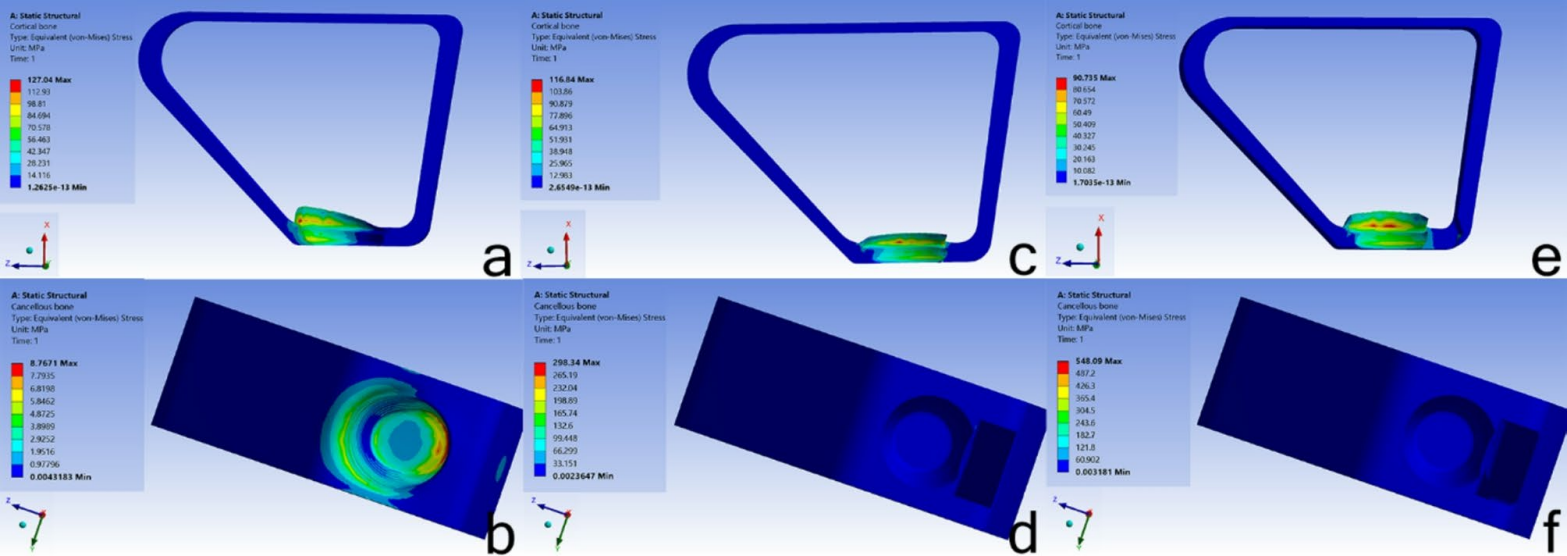
von Mises stress distribution in each model’s cortical bone, cancellous bone. MPa: Mega Pascal; Min: Minimum; Max: Maximum, **a**,** b** in HS model; **c**,** d** in BG model; **e**,** f** in SS model

In evaluating maximum principal and von Mises stress distribution within the cortical bone, the HS model exhibited the highest stress level, with a maximum of 125 MPa, 127 MPa, respectively, concentrated at the palatal cortical bone adjacent to the implant’s neck. In comparison, the SS model demonstrated the lowest stress, recording a maximum of 82 MPa and 90.7 MPa in the proximal region. The BG model also showed concentrated stress at the implant’s neck proximally, with a maximum value of 115 MPa, 116.84 MPa, respectively. Concerning the maximum principal and von Mises stress distribution in cancellous bone, the HS model again displayed the highest stress concentration, particularly at the implant’s base, with a maximum stress value of 7.6 MPa, 8.76 MPa. The BG model showed a significantly lower stress value of 3.6 MPa, 4.8 MPa, respectively, followed by the SS model of 4.3 MPa, 5.3 MPa, respectively. Figures 5 and 6.

**Fig. 7:**
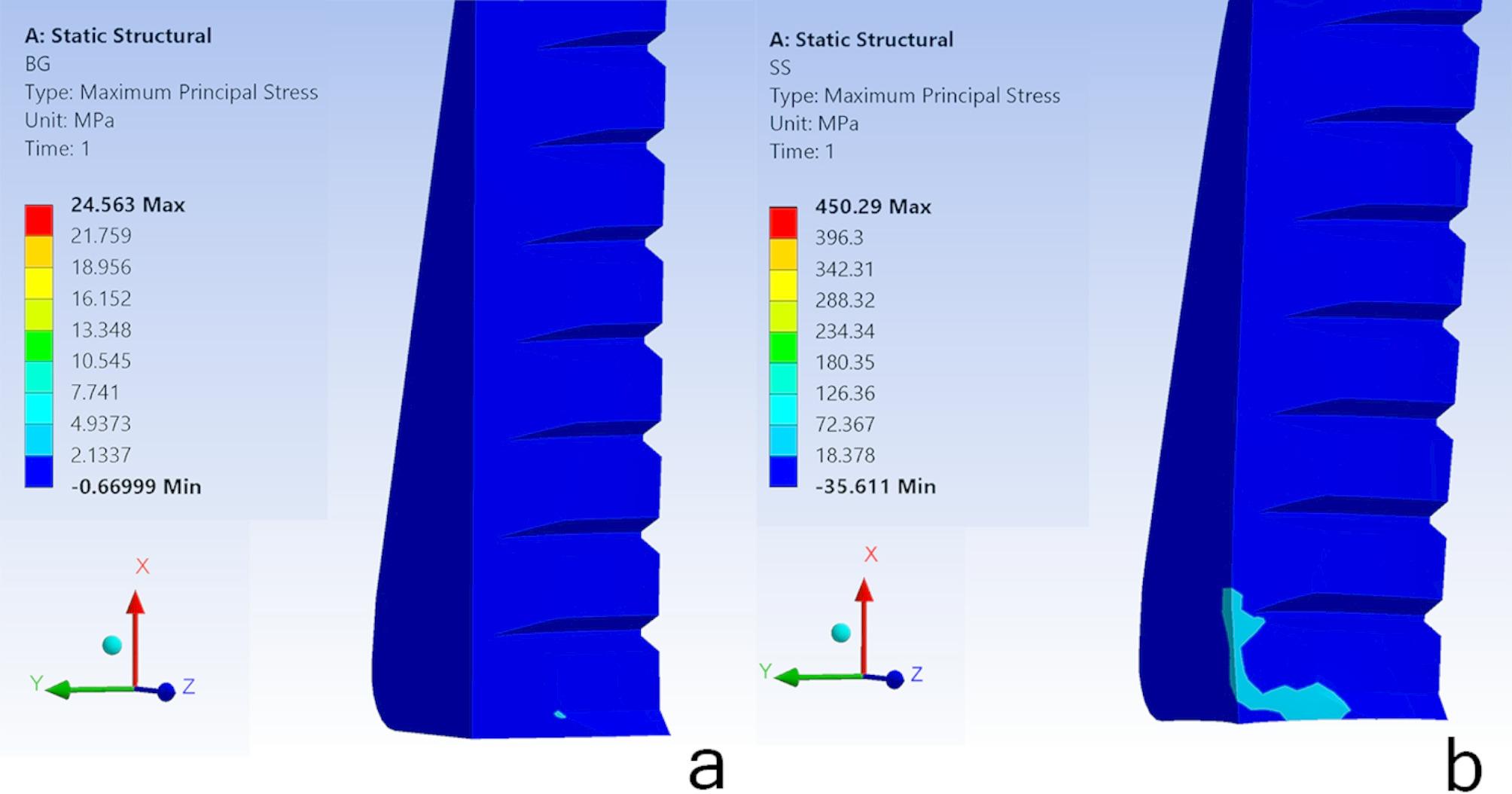
maximum principal stress distribution in SS and BG models MPa: Megapascal; Min: Minimum; Max: Maximum; **a**: Heterologous bone graft **b**: Socket shield

In the SS model, the root fragment’s maximum principal and von Mises stress were evenly distributed, measuring 28.3 MPa and 29.5 MPa, respectively. A similar uniformity was observed in the BG model, where the stress across the bone graft was measured at 2.3 MPa and 3.7 MPa, respectively. Figure 7.

**Fig. 8:**
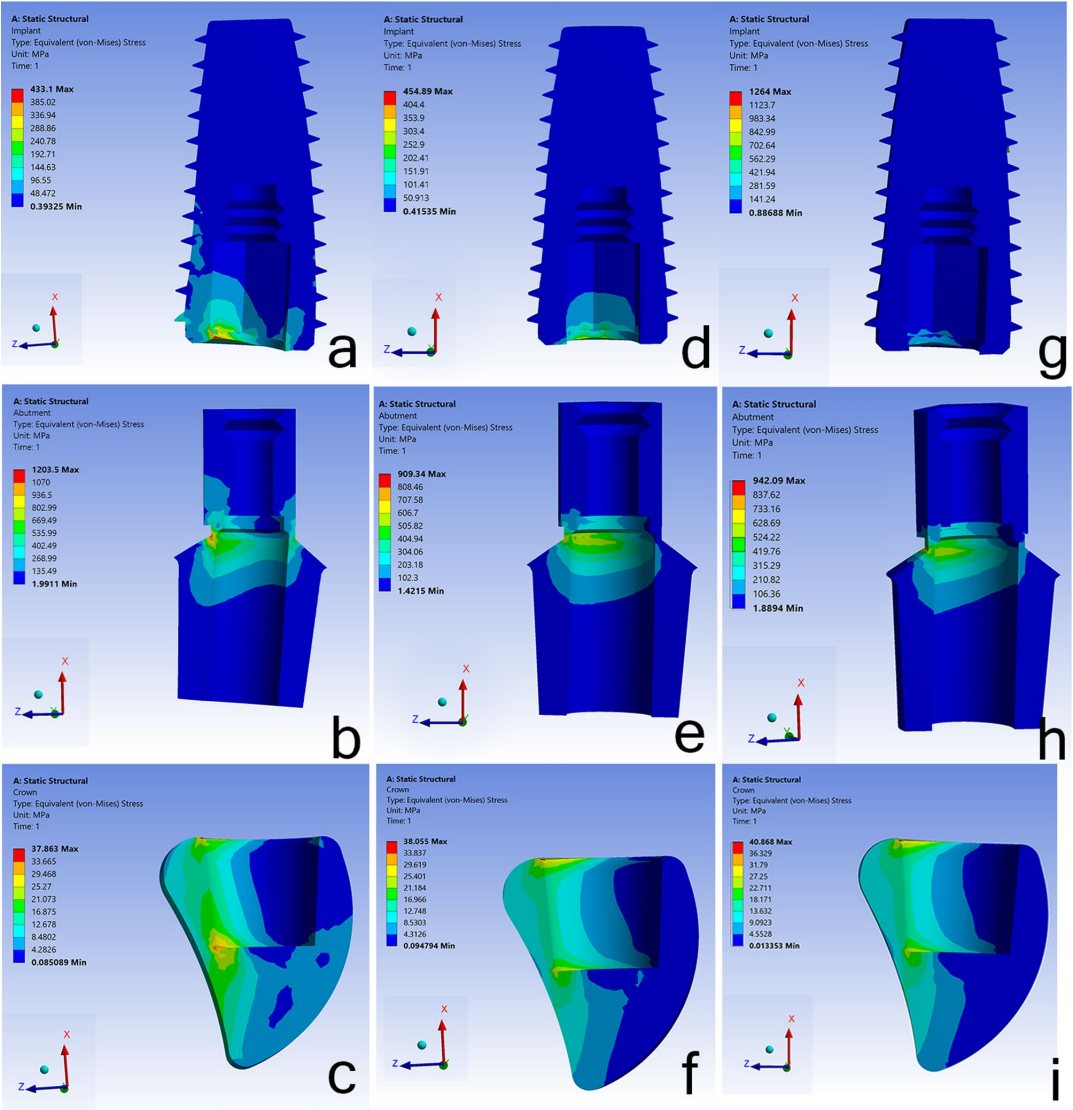
Equivalent von Mises stress distribution in implant, abutment, and crown. Min: Minimum; Max: Maximum, **a**,** b**,** c** in HS model; **d**,** e**,** f** in BG model; **g**,** h**,** i** in SS model

When assessing the von Mises stress distribution associated with the implant, the HS model recorded the highest stress value of 385 MPa, predominantly localized on the palatal side of the implant. In contrast, the BG model exhibited the lowest stress at 252 MPa, primarily on the proximal side. The SS implant also displayed significant stress concentration on the proximal side, with a value of 281 MPa. Furthermore, the von Mises stress distribution concerning the abutment revealed that the HS model exhibited the highest stress level of 936 MPa. Conversely, the BG and SS models recorded lower stress values of 505 MPa and 524 MPa, respectively. Across all models, the stresses were consistently concentrated at the abutment’s neck. Figure 8.

In the PMMA crown, the distribution of stress in the various models revealed that the highest stress level of 40 MPa occurred in the SS model at the neck and mid-palatal region of the crown. Conversely, the lowest stress levels were observed in the HS model in the same area, which closely resembled the BG model with stress values of 37 MPa and 38 MPa, respectively. Figure 8.

## Discussion

Finite element analysis (FEA) is valuable for delivering comprehensive qualitative and quantitative insights into the biomechanical behavior of dental implants. However, certain assumptions must be made to replicate realistic conditions, which can distort the model. The validity of FEA relies heavily on the geometry of the model, as well as the material properties and the conditions at the boundary and the bone-implant interface [37]. 

In this study, the CBCT data facilitated the development of anatomically representative models of the anterior maxilla at a clinical resolution, appropriate for macro-level finite element analysis, as substantiated by prior research. The finite element analysis models based on CBCT enhance anatomical realism compared to the simplified geometrical models often used in previous studies [37]. 

A prior FEA suggested that the maximum size of the root fragment for the socket shield technique should be 1.5 mm [19]. According to Mitsias et al. [38], to avoid damaging the periodontal ligament during surgery, tooth root fragments need a buccolingual thickness of at least 0.5 to 1 mm. Thin root pieces are more susceptible to breaking under the pressure of the implant during insertion [39]. Building on these prior findings, our simulation of the socket shield model (SS) assumed a root fragment thickness of 1.5 mm to replicate the optimal clinical scenario.

The mechanical properties, including Poisson’s ratios and elastic moduli for each component in the three models, were derived from either manufacturer data or earlier research. However, the properties may be average and vary in individual patients.

Due to the aesthetic demands of the anterior maxillary area, the prosthetic restoration may be provided without complete osseointegration. Thus, it is essential to simulate the imperfect contact between the dental implant and bone when assessing immediate loading under intraoral stresses. Contact interfaces with friction representing Immediate loading was defined by setting the friction coefficient to 0.3 as non-osseointegrated between Bone graft/bone, implant/bone, implant/dental fragment, and implant/bone-graft based on standard values cited in Table 3.

The periodontal ligament/alveolar bone interface was modeled as a continuous contact zone devoid of interfacial separation or slip, based on the anatomical continuity of Sharpey’s fibers and surrounding alveolar bone under initial loading conditions [19, 29]. 

In the current model, the abutment/implant and abutment/provisional crown connection were assumed to be perfectly bonded. This was done to isolate the biomechanical behavior of the implant-bone interface under immediate loading, a common approach in FEA studies [25, 26]. Intracoronal interfaces are not always ideally bonded in real clinical situations. Therefore, we suggest that future research explore different friction coefficients to reflect prosthetic interface behavior.

The load settings in a finite element model play a crucial role in the analysis. When an implant is surgically inserted into the jawbone, it is mechanically screwed into a narrower drilled hole. Significant stresses occur due to the torque applied during the insertion process. Since the stability and stress state of an immediately loaded implant can be affected by these conditions, they should also be factored into finite element simulations of such implants. In order to simulate this fact, the preload resulting from the applied tightening torque was quantified at 522 N [30]. This stress was incorporated into the analysis as the initial load step. Masticatory forces can be categorized as axial, non-axial, or a mix of both, known as mixed loading. This load simulates realistic masticatory forces, especially during anterior biting or lateral jaw movement. The force applied may be angled relative to the implant axis, allowing for component resolution in both longitudinal and transverse directions [37, 40]. This study used mixed loading with a force of 25.5 N perpendicularly to the implant’s long axis to simulate protrusive movements. Additionally, a 178 N force was applied obliquely at a 30° angle to the palatal surface of the PMMA crown, representing natural clinical conditions in the anterior region [31, 32]. However, in vivo masticatory forces may exceed this study’s oblique and horizontal forces. Therefore, further in vivo research is needed to confirm the current study’s findings.

The study demonstrated that the three implant placement techniques exhibited significantly different stress patterns and values on the peri-implant tissues, implant, and prosthetic components upon immediate loading. Therefore, the null hypothesis was rejected.

The masticatory strain that bone tissue is subjected to is critical to dental implant success [41]. Based on Newton’s third law, chewing forces applied to prosthetic restorations are converted into energy and dispersed in specific proportions throughout the implant prosthesis complex and surrounding bone tissue [42]. In immediately loaded implants, more significant stress and strain occur in the cortical and cancellous bone due to the transfer of compressive and frictional forces at the contacting interfaces, in contrast to the bonded interfaces of delayed implants [43, 44]. When under load, the implant maintains constant contact with the surrounding bone, affecting both the remodeling and repair of bone tissue and its destruction, depending on the direction and intensity of the load. The alveolar bone tissue has a pressure-stress regulation mechanism. When the load reaches a certain threshold, the bone tissue initiates the osteogenic process, strengthening the bone structure and decreasing the unit tissue strain rate. Conversely, an insufficient load causes bone resorption and weakening. Thus, immediate loading following immediate implant placement aids in restoring masticatory stress to the alveolar bone, consequently preserving the alveolar bone [45]. 

In the SS model, the cortical bone’s maximum principal and von Mises values were significantly lower than in other models (82 MPa), (90.73 MPa), respectively. This may be explained by the Periodontal Ligament (PDL), which can substantially reduce the stresses on the trabecular bone and the root fragment with its minimal [46]. The PDL attachment with the cementum and alveolar bone creates fibrous entheses that can transmit mechanical stress from the root fragment to the alveolar bone and exhibit excellent load-transmitting characteristics [47]. Additionally, the PDL has neurovascular components that support the relative motion between the fragment and the bone and distribute cyclic masticatory forces, resulting in short-term continuous adaptation of the bone-PDL-cementum complex [48]. 

Conversely, the HS model had the highest maximum principal and von Mises values in the cortical bone (125 MPa), (127Mpa), respectively. The greater contact area between the implant and bone tissue might explain this finding. The cortical bone can withstand greater loads, has a higher modulus of elasticity, can bear more load, and is more deformation-resistant than trabecular bone [49]. 

The stress distribution pattern of cortical bone in the SS and BG models was concentrated in the proximal area around the neck of the implant. In contrast, the HS model showed that stress was primarily concentrated on the palatal side, also near the neck of the implant. This difference can be explained by the fact that, in the SS and BG models, the diameter of the implant was smaller than the size of the extraction wound following tooth extraction. As a result, the buccal and palatal areas of the cortical bone didn’t make direct contact with the implant’s threads, so stresses were concentrated more proximally. However, in the HS model, the distance between the cortical bone and the implant on the palatal side was much closer, resulting in stress concentration palatally.

The maximum principal and von Mises values of cancellous bone showed a significant lower value across the BG model (3.6 MPa), (4.8 MPa), respectively and the SS model (4.3 MPa), (5.3 MPa), respectively; This may be explained by the presence of bone graft and root fragment in the BG and the SS models, which have a low modulus of elasticity, leading to better dissipation of chewing forces to the cancellous bone. However, the HS model showed the highest value (7.6 MPa), (8.76 MPa), respectively. As the implant is mechanically screwed into a narrower drilled hole, the stress distribution pattern is mainly concentrated at the base of the implant. In contrast to cortical bone, cancellous bone’s lower elastic modulus serves as a stress reliever, resulting in reduced load transmission through it [50]. 

The maximum principal and von Mises values of the bone graft in the BG model (2.3 MPa), (3.7 MPa), respectively, showed a significant difference from the root fragment in the SS model (28.3 MPa), (29.5 MPa). The comparable mechanical characteristics of the bone graft and root fragment could explain the different results. However, histological evidence demonstrates that the root fragment simulated in the SS model maintains its characteristics. In contrast, the bone graft simulated in the BG model will likely alter over time due to bone maturation [51, 52]. 

In this study, PMMA was used as a provisional restoration, considered an intermediate stage for short- or long-term placement on dental implants between the surgical installation of the implant and the fabrication and placement of definitive restorations once the implant has wholly osseointegrated [53]. PMMA crown was used as a provisional restoration due to its shock-damping behavior, favorable resilience, and low modulus of elasticity. Further investigation showed that the stress values recorded in the artificial crown typically declined as its material stiffness decreased [54]. This could explain the similar stress distribution patterns, mainly concentrated at the neck and mid-palatal region of the crown in the three models. Previous research has clearly shown that changes in crown stiffness do not significantly impact peri-implant stress values during noncantilever loading. This finding is crucial for ensuring the longevity and effectiveness of implants, as it emphasizes that crown stiffness variations are not a primary concern in these scenarios and do not demonstrate significant effects on the peri-implant stress values under non-cantilever loading [55, 56]. 

The abutment’s von Mises stress value showed no significant difference across the BG (505 MPa) and the SS (524 MPa) models; however, the HS model presented the highest value (936 MPa). The three models showed similar stress distribution patterns concentrated mainly at the neck of the abutment. The implant’s von Mises stress value showed a significant difference between the three models, the BG and SS models recorded lower stress values of 252 MPa and 281 MPa, respectively, while The highest value was presented in the HS model (385 MPa); nevertheless, the stress distribution pattern was mainly concentrated at the implant’s neck in the three models, similar to previous studies [17, 53]. 

A prior study indicated that any von Mises stress exceeding the yield strength of a titanium implant (550 MPa) could lead to failure [17]. In the current study, none of the implants demonstrated a von Mises stress value above 550 MPa. However, when the Hs model was immediately loaded, the stress on the titanium abutment reached 936 MPa, surpassing the 550 MPa threshold. This finding suggests that the abutment is at a greater risk of failure (such as loosening or fracture) in immediate loading scenarios, as the stress was concentrated at the abutment’s margin. Additionally, the maximum principal and von Mises values across all models remained below the ultimate strength limits of cortical bone and fell within the ultimate strength range of trabecular bone. Nevertheless, the SS model’s stress value on the cortical bone demonstrated a notable variation across the three models. This decreased value might indicate reduced alveolar bone resorption and improved aesthetic results.

The present study offers innovative insights into the mechanical performance of immediate implant protocols within the esthetic zone. Although no prior research has directly examined FEA of immediate implants protocols, a previous randomized clinical trial evaluated the Socket shield technique against conventional immediate implant placement with immediate temporization, revealing that both groups achieved a short-term implant success rate of 100%. However, the study indicated that patients who underwent the modified shield technique experienced a higher Pink Esthetic Score and greater satisfaction than those who received immediate implant placement [57]. Another randomized controlled clinical trial examined the dimensional changes in soft and hard peri-implant tissues around single immediate implants in the esthetic zone, comparing the socket shield technique with xenograft use. Results showed that the socket shield group experienced significantly less vertical and horizontal buccal bone resorption than the xenograft group. Additionally, the xenograft group had considerably more midfacial mucosal recession than the midfacial mucosal coronal migration observed in the socket shield group [58]. A prior systematic review and meta-analysis of the socket shield technique for immediate implant placement found that, in the short term, the SS helps minimize changes in the buccal plate’s width and height while enhancing the soft tissue profile after immediate implant placement in the esthetic zone [59]. 

While our FEA models offer high anatomical and mechanical fidelity, several limitations must be acknowledged in light of existing literature. In this study, we assumed the simulated materials were linearly elastic, isotropic, and homogeneous. We adopted these assumptions from previous FEA studies for consistency and ease of calculation. While this model’s simplifications promote reproducibility, they also limit generalizability to diverse patient anatomies and clinical conditions. We acknowledge that biological tissues exhibit anisotropic, heterogenous, and viscoelastic behavior, which affects stress distribution over time. Although these simplifications are common in FEA literature, they reduce the model’s ability to predict long-term biomechanical outcomes.

This analysis primarily examines bone conditions (HS, BG, and SS) with single-diameter implants. However, factors related to implant design—such as diameter, length, and thread geometry—could affect stress distribution. As a result, additional research is essential to explore the biomechanical behavior of different implant parameters.

Although the SS technique showed favorable stress distribution in this simulation, long-term histological and clinical data are needed to confirm whether these mechanical benefits result in improved implant survival and aesthetic outcomes. While the literature supports the SS technique for short-term aesthetic benefits [57–59], long-term randomized controlled trials remain limited.

## Conclusion

Within the limitations of this study, several important conclusions can be drawn.

1. FEA highlighted the significance of applying biomechanical principles and understanding alveolar bone response to optimize implant treatment strategies.
2. The socket shield technique exhibits advantageous biomechanical performance under immediate loading conditions by reducing stress on peri-implant bone and implant components. These results endorse its clinical applicability but necessitate further in vivo validation.

## Data Availability

All data generated or analysed during this study are included in this published article.

## Abbreviations

°C: Centigrade Degree
BG: Bone Graft
BIC: Bone-Implant Interface
CAD: Computer-Aided Design
CBCT: Cone Beam Computed Tomography
FEA: Finite Element Analysis
HS: Healed Socket
Mm: Millimeter
MPa: Mega Pascal
N: Newton
PDL: Periodontal Ligament
PMMA: Poly Methyl Meth Acrylate
SS: Socket Shield
STL: Standard Tessellation Language
